# Fetal Cardiac Interventions—Polish Experience from “Zero” to the Third World Largest Program

**DOI:** 10.3390/jcm9092888

**Published:** 2020-09-07

**Authors:** Marzena Debska, Adam Kolesnik, Beata Rebizant, Agnieszka Sekowska, Agnieszka Grzyb, Katarzyna Chaberek, Jacek Witwicki, Romuald Debski, Joanna Dangel

**Affiliations:** 1 2nd Department of Obstetrics and Gynecology, Centre of Postgraduate Medical Education, 01-809 Warsaw, Poland; drsekowska@gmail.com (A.S.); chaberek.katarzyna@gmail.com (K.C.); aldek@post.pl (R.D.); 2 Department of Gynecologic Oncology and Obstetrics, Centre of Postgraduate Medical Education, 00-416 Warsaw, Poland; 3 Department of Perinatal Cardiology and Congenital Anomalies, Centre of Postgraduate Medical Education, US Clinic Agatowa, 03-680 Warsaw, Poland; akolesnik@cmkp.edu.pl (A.K.); agagrzyb@gmail.com (A.G.); jdangel@cmkp.edu.pl (J.D.); 4 Cardiovascular Interventions Laboratory, The Children’s Memorial Health Institute, 04-730 Warsaw, Poland; 5 Department of Descriptive and Clinical Anatomy, Medical University of Warsaw, 02-004 Warsaw, Poland; 6 Pain Clinic, Department of Anesthesiology and Intensive Care, Centre of Postgraduate Medical Education, 00-416 Warsaw, Poland; 7 Department of Cardiology, The Children’s Memorial Health Institute, 04-730 Warsaw, Poland; 8 Department of Neonatology, Centre of Postgraduate Medical Education, 01-809 Warsaw, Poland; jacek.witwicki@bielanski.med.pl

**Keywords:** fetal cardiac interventions, fetal valvuloplasty, fetal echocardiography, critical aortic stenosis, hypoplastic left heart syndrome, pulmonary atresia and intact ventricular septum, technical aspects

## Abstract

This article presents the technical aspects of the Polish fetal cardiac interventions (FCI) program, including preparation of the team and modifications in the technique of the procedure that aim to increase its safety for the mother and the fetus. Over 9 years, 128 FCI in 113 fetuses have been performed: 94 balloon aortic valvuloplasties (fBAV), 14 balloon atrioseptoplasties (fBAS) with stent (BAS+), 5 balloon atrioseptoplasties without stent placement (BAS−), and 15 fetal pulmonary valvuloplasties (fBPS). The technical success rate ranged from 80% (BAS−) to 89% (fBAV), while the procedure-related death rate (defined as death within 72 hours following the procedure) ranged from 7% (fBAV and fBPV) to 20% (BAS). There were 98 live births after all FCI (3 pregnancies continue). Median gestational age at delivery was 39 weeks in our center and 38 weeks in other centers.

## 1. Introduction

Fetal cardiac interventions (FCI), although still considered an experimental therapy [1], are being performed in an increasing number of centers around the world. There are two main goals of FCI: the first is to change the prenatal history of the critical aortic stenosis (AS) and pulmonary atresia with intact ventricular septum (PA&IVS), both of which when untreated could evolve to a functionally univentricular heart. The second is to improve survival in fetuses with closed foramen ovale and hypoplastic left heart syndrome (HLHS), or critical AS with severe heart failure or dysplastic mitral valve syndrome. Our program was launched in June 2011, following the visit to a leading center for fetal cardiac interventions in Europe [2] and relatively soon became one of the largest in the world. We present the establishment and evolution of our FCI program, analyze the technical aspects of the interventions, and try to define factors that turned out to be crucial for the technical success.

## 2. Methods

**Figure 1:**
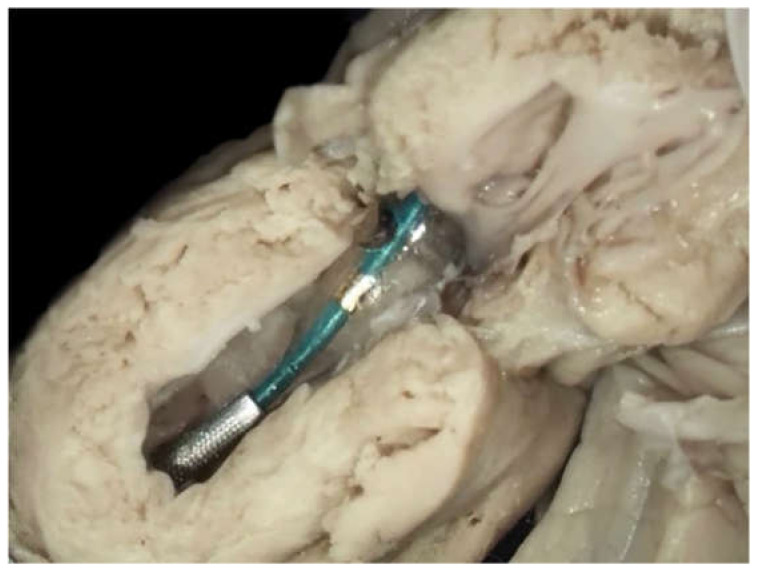
Training on the cardiac specimen in the dissection room. The catheter was introduced through the apex into the heart of the neonate with critical aortic stenosis. The guidewire with a balloon catheter was introduced through the left ventricular apex and the aortic valve.

**Figure 2:**
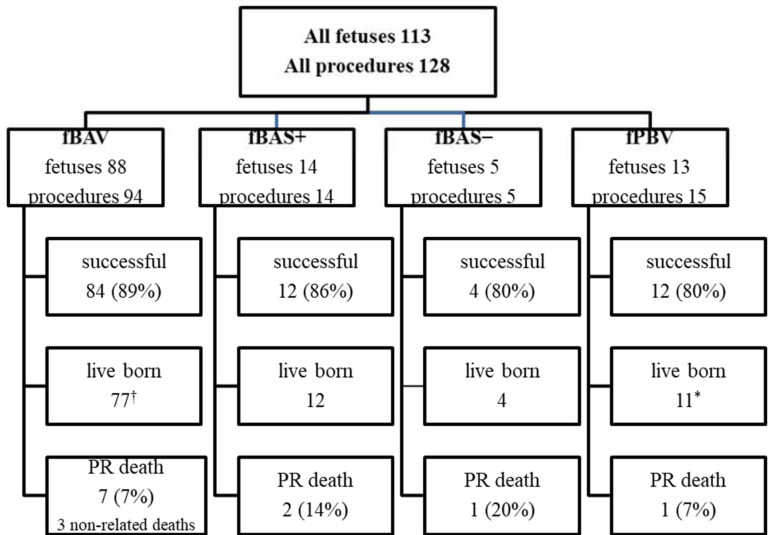
Fetal cardiac interventional procedures performed between 2011 and 2020. fBAV: fetal balloon aortic valvuloplasty; fBAS+: stent placement in the interatrial septum; fBAS−: balloon atrioseptoplasty; fBPV: fetal balloon pulmonary valvuloplasty; PR death: procedure-related death. A total of 14 fetuses had more than one procedure (8 fetuses had the same procedure twice, 5 fetuses had two different procedures, and 1 fetus had three different procedures). † two pregnancies continue; * one pregnancy continues. Eight fetuses with critical aortic stenosis were hydropic, and six of them needed opening of the interatrial septum after fBAV.

Before starting the procedures, the team of specialists, including a fetal cardiologist (J.D.), a feto-maternal specialist (M.D.), and an interventional cardiologist (A.K.), was formed and the preliminary protocol was prepared. Having observed cardiac interventions in another center, the whole group took part in the procedures in the dissecting room on a cardiac specimen (Figure 1). This training was undertaken to check the proportion between fetal heart structures and used equipment. The program was approved by the Ethical Committee of the Centre of Medical Postgraduate Education and offered for all patients at no cost as a part of national health insurance. Data of FCI were gathered prospectively since the beginning of the program. All fetuses underwent echocardiography with detailed structural and hemodynamic assessment in the Department of Perinatal Cardiology and Congenital Anomalies, Centre of Postgraduate Medical Education, US Clinic Agatowa, and the procedures were performed in the 2nd Department of Obstetrics and Gynecology of the Centre of Postgraduate Medical Education in Bielanski Hospital, Warsaw. The computer database of the results of fetal echocardiography exams and records of all fetal interventions from June 2011 to March 2020 were reviewed. All fetuses who underwent FCI were included in this study (Figure 2). We analyzed the maternal and fetal anesthesia and technical aspects of consecutive procedures, ways in which we dealt with arising complications that were crucial for the safety and success of the procedure, and issues that could lead to failure or complications of FCI (Table 1).

Fetuses for FCI were recruited on the basis of the international criteria [3,4,5]. Following a detailed fetal echocardiography, parents were initially counselled by the fetal cardiology specialist. After being admitted to the hospital, the mother was examined by an obstetrician and signed an informed consent form and agreement for the experimental therapy. There were no mothers’ contraindications to the procedure; one procedure was delayed for one week because the pregnant woman was treated with acetylsalicylic acid for obstetric reasons. In 6 cases we withdrew from the procedure (in 4 cases due to persistent unfavorable fetal position, in 2 cases mother resigned after giving initial consent).

All fetal interventions were performed by the same team: the fetal cardiologist (J.D.), the feto-maternal specialist (M.D.), the interventional cardiologist (A.K.). The ultrasound machine during the procedure was operated by another obstetrician (B.R.), who also took care of patients before and after the procedure. The operating theatre team consisted of an anesthesiologist, an anesthesiology nurse and a scrub nurse.

The protocol on preparing pregnant women for the fetal intervention was established. 

The equipment for interventional procedures: needles, guidewires, balloon catheters and stents, were prepared based on their current availability in Poland. (File S1)

All procedures were performed in an antiseptic environment of the operating theatre, percutaneously under ultrasound guidance. The surgical site was prepared by applying an antiseptic solution and then covering with surgical plastic drapes. Then the ultrasound probe was covered by a sterile protection sleeve and sterile ultrasound gel was applied. The mother was placed on her back, or slightly on her left or right side, depending on the fetal position. If the fetal position was unfavorable, the team tried to change it before the procedure asking the mother to do certain exercises. Sometimes external manual maneuvers were performed on the fetus. The procedure was started if the position of the fetus was optimal or close to optimal, and it was eventually possible to correct it by moving the fetus with the needle introduced into the uterus.

Basing on the data gathered from international publications [6] we performed our first 8 procedures (7 aortic valvuloplasties and 1 balloon atrioseptoplasty) under general anesthesia of the mother while the fetus was indirectly anesthetized by medications (fentanyl and midazolam) crossing the placenta.Before each procedure cordocentesis was performed to take 4 mL fetal blood sample for NTproBNP, CRP, procalcitonin concentration and genetic tests to diagnose possible coexisting problems in the fetus. Beginning from the 9th procedure we replaced fetal transplacental anesthesia by giving fentanyl and atracurium directly into the umbilical vein. From the 32nd procedure atropine was added before the procedure aiming to decrease the risk of fetal bradycardia.

**Figure 3:**
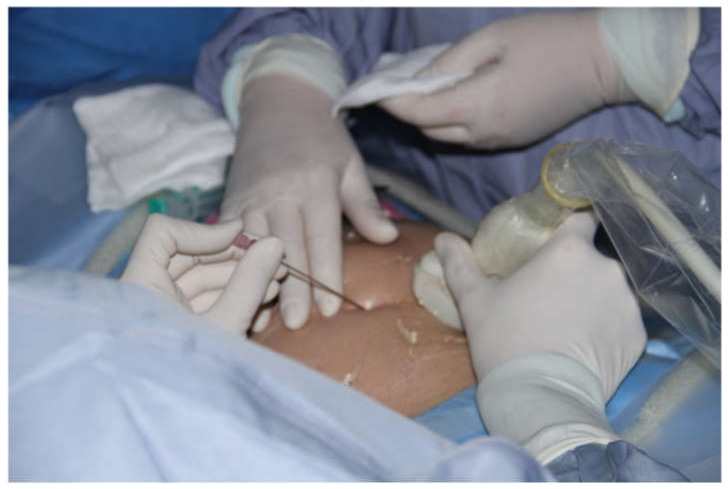
The technique of performing all cardiac interventional procedures. Antiseptic operating field, transducer covered by antiseptic sleeve. The obstetrician holds the transducer in the right hand and the needle in the left hand.

Before introducing the 18G needle the mother’s skin was additionally anesthetized with local lidocaine. The obstetrician performed the fetal blood sampling (FBS) and cardiac puncture with the left hand, keeping the ultrasound probe in the right hand to monitor the procedure. (Figure 3) To achieve the perfect angle for insertion of the needle, the obstetrician if needed corrected slightly fetal position with the 18 G needle (after she withdrew the tip of the trocar to avoid injury of the fetus). In 3 cases two 18 G needles were used during one procedure - one was necessary to stabilize fetal position and the other to puncture fetal heart. In 3 cases interventions were cancelled after mother and fetal anesthesia because the fetuses changed in the meantime their positions significantly, thus safe and successful operation was not possible.

The equipment for cardiac interventions was prepared by the cardiologists. The optimal balloon size was chosen on the basis of the type of intervention and valve annulus diameter measured directly before the procedure. In the majority of cases, the fetal heart was punctured with a 18 G needle; a 17 G needle was used only when a 4.5 mm or 5 mm balloon or a stent larger than 4 mm was used. In such cases, using a Y-connector was necessary to avoid fetal blood loss through the space between the catheter and the needle. A coronary guidewire was introduced through the needle; it was cut before the intervention to obtain the appropriate length for manipulation. A torquer was put on it to improve guidewire handling.

Guidewires and catheters were washed with 0.9% NaCl solution before insertion into the needle for a better slip. Beginning with the 32nd procedure, we started to add standard heparin (UFH) to this solution, aiming to decrease the risk of clotting formation during manipulation within the cardiac cavities. From the 36th procedure, we proceeded to rapidly evacuate even a small amount of pericardial effusion; since it was the most common cause of fetal bradycardia, we did not wait until bradycardia occurred.

After each procedure, the fetus was monitored with ultrasound for 60 minutes in the ultrasound laboratory, and if everything was normal, the patient was transferred to the postoperative ward. The fetus was then monitored by cardiotocography or Doppler ultrasonic fetal heart detector, depending on pregnancy stage. Antibiotic was given routinely for 7 days. After aortic and pulmonary valvuloplasty, digoxin was administered to the mother to improve fetal cardiac function and it was continued until delivery.

**Figure 4:**
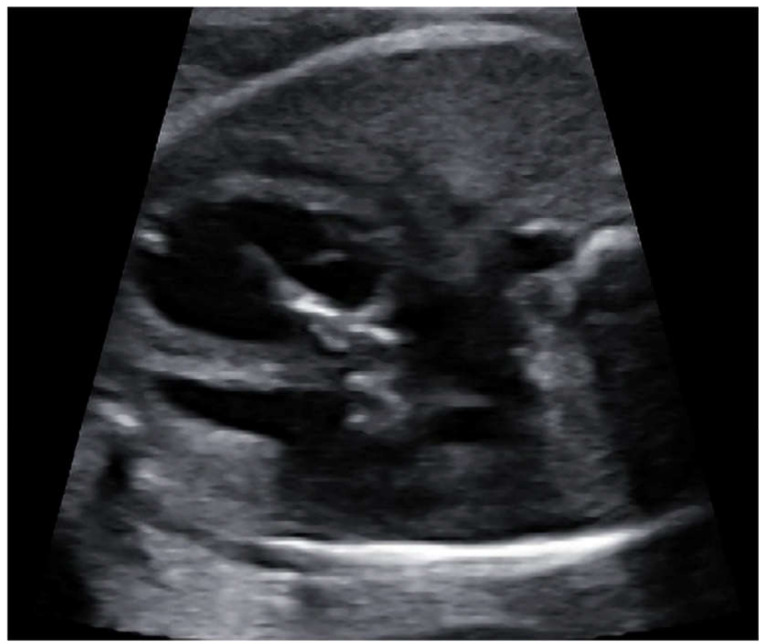
fBAV—the coronary balloon inflated in the aortic valve. The left ventricular apex was punctured and the needle with the coronary balloon was introduced through the aortic valve.

Fetal balloon aortic valvuloplasty (fBAV) was considered successful when the balloon crossed the aortic valve, was inflated and deflated at least three times, and forward flow through the aortic valve was larger than before the procedure (Figure 4). Aortic insufficiency was the additional sign that the valve was dilated, however, its appearance was not necessary to consider the procedure successful. 

**Figure 5:**
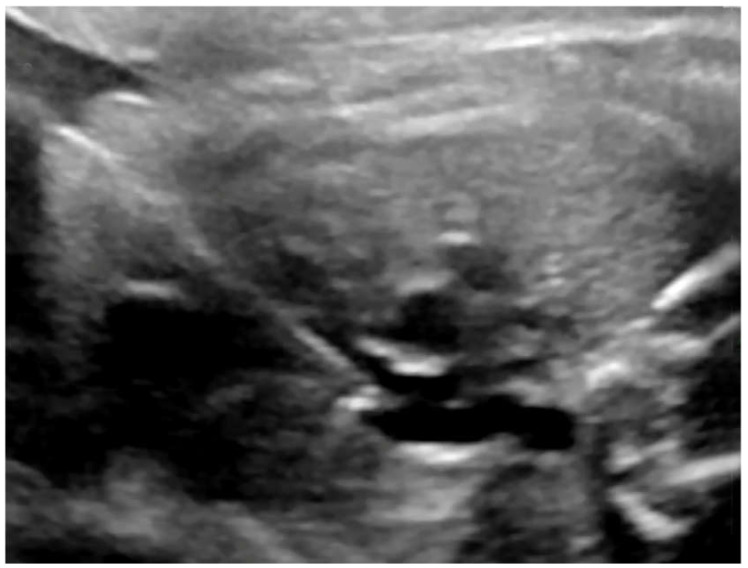
fBPV—the guidewire was introduced through the needle and placed across the pulmonary valve.

Fetal balloon pulmonary valvuloplasty (fBPV) was considered successful when the pulmonary valve was perforated with the needle and the guidewire with a balloon was introduced to the pulmonary trunk, the balloon was inflated and deflated at least three times, and the forward flow through the pulmonary valve was larger than before the procedure (Figure 5).

Fetal balloon atrioseptoplasty (fBAS−) was considered successful when the interatrial septum was punctured with a needle, the balloon was introduced and inflated at least once within the interatrial septum, and left to right flow was visible in the color Doppler image. 

Fetal stent placement (fBAS+) in the interatrial septum was considered successful when the interatrial septum was punctured with a needle, the balloon with a stent was introduced into the left atrium, the stent was placed in a proper position within the septum, and left to right flow through the stent was visible in the color Doppler image.

## 3. Results

### 3.1. All Procedures—Technical Results

Between 6 June 2011 and 30 April 2020, the team performed 128 FCI in 113 fetuses: 94 balloon aortic valvuloplasties (fBAV) in 88 fetuses, 15 pulmonary valvuloplasties (fBPV) in 13 fetuses, 5 balloon atrioseptostoplasties (fBAS-) in 5 fetuses, and 14 stent placements into the interatrial septum (fBAS+) in 14 fetuses (Figure 2). In six fetuses with critical aortic stenosis and severe heart failure, both fBAV and interatrial opening was necessary. In one of them, three procedures were performed: fBAV, balloon atrioseptoplasty, and stent placement into the interatrial septum. 

**Figure 6:**
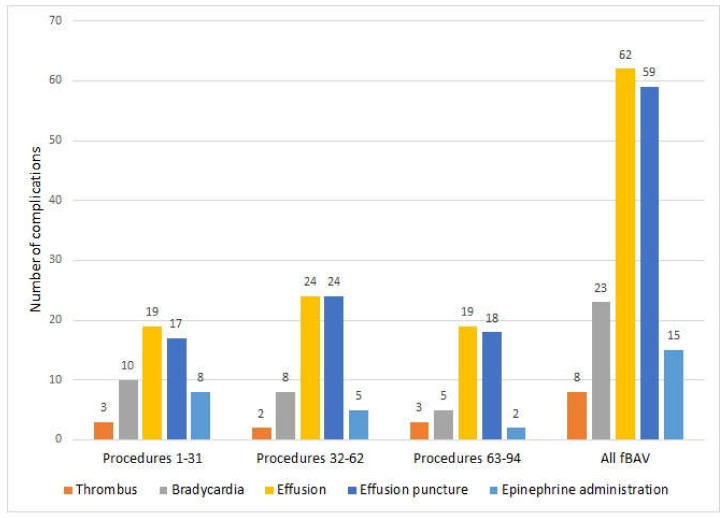
Most common complications during fBAV. Procedures were divided into three parts, with around 30 fBAV in each. From the 32nd procedure, we started to add heparin into the flushing solution and atropine into the umbilical vein. From the 36th procedure, we started to evacuate even a small amount of pericardial effusion.

The most common complication after the FCI procedures was pericardial effusion, which usually occurred after the needle withdrawal from the heart. The complication correlated with fetal bradycardia, which was present in 20 out of 62 fetuses with pericardial effusion and in 3 out of 30 fetuses without pericardial effusion (*p* = 0.022). Fetal bradycardia was statistically more frequent with fBPV (8 in 15 procedures) than fBAV (23 in 94 procedures), *p* = 0.03, while the frequency of pericardial effusion was similar between those two groups (10 in 15 procedures of fBPV vs. 62 in 94 procedures of fBAV), and it occurred in about 66% of cases. In terms of fBPV, the mean amount of effusion was significantly larger than in fBAV (12 vs. 4.7), *p* = 0.0004 (Figure 6).

For all procedures, if the fetus died within 72 hours after the procedure, we qualified it as a procedure-related death.

There were no complications after any cordocentesis. Tocolytic drugs were not routinely given before, during, or after interventional procedures. There were no preterm premature rupture of membranes (PPROMs) related to the procedure (defined as PPROM within 10 days after the FCI).

There were no terminations of pregnancy (TOP) in the treated group, even when the procedure proved unsuccessful.

### 3.2. Fetal Balloon Aortic Valvuloplasty (fBAV)

Fetal balloon aortic valvuloplasty for critical aortic stenosis (AS) was the most common fetal cardiac intervention (Video S1). A total of 94 procedures were performed in 88 fetuses. International criteria for fetal intervention were followed: patent but critically stenotic aortic valve, severely impaired left ventricle (LV) function with abnormal mitral valve (MV) inflow, LV length more than (−2) Z-score [7]. Median gestational age at the first procedure was 25 weeks of gestation and ranged from 20 to 32 weeks. There were two groups of fetuses with AS: those with evolving HLHS and with AS and severe heart failure, including eight with dysplastic mitral valve syndrome. 8 fetuses with AS were hydropic. In 5 cases the hydrops resolved after the procedure (4 cases after fBAV, and 1 case after fBAV and BAS+).

#### Technical Results of fBAV

fBAV was successful in 84 out of 94 procedures. The success rate was 89%. There were 79 live born babies with a median age of delivery of 39 weeks., with two pregnancies ongoingA total of 62babies were born in our center, and the rate of cesarean section (CS) was 40% (25 out of 62), which is a typical rate for our hospital. The cesarean sections were performed due to obstetric reasons. If the babies were born in other hospitals, the rate of CS was 67% (10 out of 15). There were no cases of procedure-related preterm premature rupture of membranes (PPROM) after any fBAV. Procedure-related death) occurred in seven fetuses (7.4%), and the last PR death was in the 49th procedure in 2015 (File S2). Minor complications (fetal bradycardia, thrombus formation, pericardial effusion, and cardiac tamponade, as well as necessity of adrenaline administration) are presented in Table 1 and Figure 6.

### 3.3. Opening the Interatrial Septum

Two types of procedures of the opening of the interatrial septum were performed: balloon atrioseptoplasty (fBAS−) and stent placement (fBAS+) (File S3).

Opening of the interatrial septum was performed to improve fetal or neonatal condition, according to published standards. 19 procedures were performed:10 in HLHS and closed interatrial septum (in all cases there was bidirectional flow in the pulmonary veins), 8 in severe aortic stenosis with heart failure and secondary closure of the interatrial septum, and 1 in Shone’s syndrome (complex defect of the left side of the heart). In five fetuses with AS and severe heart failure due to secondary closed IAS, whose condition did not improve after successful dilatation of the aortic valve, we performed opening of the interatrial septum after the fBAV procedure. In one fetus, opening the septum was performed before fBAV. 

#### 3.3.1. Balloon Atrioseptoplasty

Balloon atrioseptoplasty was performed in five fetuses, four with HLHS between 22 and 24 weeks, and one with critical AS after fBAV in 29 weeks. Four (out of five) procedures of fBAS− were successful, but it allowed effective interatrial communication only in one fetus with thin foramen ovale flap. In other fetuses, there was no real septum primum visible; the septum was very thick and it closed spontaneously soon after the procedure. With growing experience, we decided to put stents in all situations when it was technically possible. 

There was one procedure-related death (after our first fBAS−), and four babies were live born. Two deliveries were in 29 weeks of gestation (caused by polyhydramnios), and two were term pregnancies (38 and 39 weeks).

#### 3.3.2. Interatrial Stent Placement

Interatrial stent placement was performed in 14 fetuses with closed interatrial septum (IAS): 10 with HLHS and 4 with critical AS (Video S2). The procedures were performed between 22 and 34 weeks of pregnancy, with a median of 27 weeks. The procedure was successful in 12 fetuses (86%). There were two procedure-related deaths. The pulmonary venous flow improved significantly in all cases. Seven children were delivered at term, in good general condition. They were breathing spontaneously and did not have any signs of respiratory failure. Five babies were born prematurely.

Details of technique and the results of both procedures are presented in Supplementary Files. (Supplementary File—clip stent).

### 3.4. Fetal Balloon Pulmonary Valvuloplasty (fBPV)

Fetal balloon pulmonary valvuloplasty (fBPV) was performed in 13 fetuses: 8 with pulmonary atresia and intact ventricular septum (AP&IVS) and 5 with critical pulmonary valve stenosis with left to right shunt across the ductus arteriosus and severely impaired, small right ventricle. The size of pulmonary valve annulus was within normal limits in all cases, and tricuspid valve z-score was at the lower normal limits. The team followed the described indications [8] and modified them according to their own experience and to the results of treatment of children after birth. If there was any doubt if the fetus actually needed an intervention, the team waited for 1–2 weeks to be certain that the RV function deteriorated.

fBPV was the most difficult procedure for the team (File S4), as the right ventricular outflow tract (RVOT), particularly in smaller pregnancies, was sometimes very short and close to the mediastinum. It was difficult to put the needle in a proper position to achieve enough space to release and then safely inflate the balloon without damaging the wall of the ventricle. In the case of pulmonary valve atresia, the often thick and stiff valve had to be perforated by the needle that additionally created the risk of pulmonary artery wall injury. RVOT proved to be very sensitive and manipulating in it with a needle could easily cause fetal bradycardia.

#### Technical Results of fBPV

fBPV was performed between 22 and 30 weeks of gestation, with a median of 24 weeks. It was successful in 12 out of 15 procedures (80%). If fBPV was successful, the right ventricle kept growing towards the end of pregnancy. The most common complication was fetal bradycardia, which occurred in 8 out of 15 procedures (53%). In one fetus, a transient complete heart block appeared, which was successfully treated with intracardiac adrenaline administration. There were 11 live births (one pregnancy continues), and one fetus was delivered prematurely outside the reference center due to obstetrical problems. Median gestational age at delivery at our center was 39 weeks (Video S3).

### 3.5. Methodological Modifications

#### 3.5.1. Anesthesia

After initial eight interventions we realized that in most cases only two or three needle punctures were required to complete the procedure. In such circumstances, general anesthesia with the intubation of the pregnant woman seemed to bring an unnecessary and excessive risk when the fetus could be separately and more efficiently anesthetized and immobilized with medications given directly into the umbilical vein. Since then, general anesthesia of the mother was permanently and successfully replaced with conscious analgosedation using fentanyl and midazolam; the mother was responsive and conscious during the procedure. General anesthesia was necessary only in three patients in whom, due to prolonged fetal resuscitation, time of the procedure exceeded the generally accepted limit for this type of anesthesia. The mothers’ condition improved significantly with analgosedation. They did not experience unpleasant symptoms after intubation and high doses of anesthetic drugs and they quickly returned to normal activity.

Direct anesthesia of the fetus with the addition of atracurium (a paralyzing drug that insufficiently crosses the placenta) effectively anesthetized and immobilized the fetus during the procedure.

Some fetuses were very active and often changed position before the procedure. In 22 such cases, fentanyl was administered intramuscularly (IM) to the fetal buttock or thigh to decrease their mobility and prevent them from changing their position. In 10 cases, it was performed in the ultrasound laboratory before transporting the mother to the operating theatre, and in 10 cases in the operating theatre before cordocentesis. The remaining dose of medications was then given through the umbilical vein in the operating theatre. In two cases, cordocentesis could not be performed due to very thin umbilical vein, and those fetuses were anesthetized via intramuscular injection only.

#### 3.5.2. Heparin

Addition of heparin to the fluid in which we flushed the catheters did not appear to have any protective effect on the appearance of intracardiac thrombus, as well as any effect of bleeding complications. However, in the subjective perception of the operators, the manipulation with the catheter and the balloon inside appeared smoother.

#### 3.5.3. Atropine and Early Pericardial Drainage

To prevent fetal bradycardia, we at the same time started to add atropine to the set of medications used for fetal anesthesia and proceeded to rapidly evacuate even a small amount of pericardial effusion without waiting for the fetal heart rate decrease to occur. Before this modification in the group of fBAV, there was bradycardia in 10 out of 31 fetuses, compared with 13 in out of 63 fetuses after this modification. Although this difference was not statistically significant for the whole group (*p* = 0.196), if we divided all fBAV procedures into three time groups, there was a significant difference between the group 1 to 31 procedures and the group 63 to 94 procedures regarding adrenaline administration (8 in 31 cases in the first group and 2 in 32 in the last group, *p* = 0.037 (Figure 6)).

## 4. Discussion

In terms of the number of patients, the most experienced centers in the world are in Boston and Linz, and our program is the third largest. According to the available literature, in Boston 136 fBAV [9] and in Linz 92 fBAV [10] and 35 fBPV [11] have been performed. Some data regarding FCI are collected in the International Fetal Cardiac Intervention Registry (IFCIR) [1].

Like in the other centers, the most common FCI procedure was fBAV (94) but in contraty the second most common procedure was interatrial septum opening (19) the third one was fBPV (15). In our series, the most serious complication—procedure-related fetal death—occurred in 8% of cases (10 cases out of all 128 procedures). In our material, fBAV and fBPV carried a similar complication rate, with a procedure-related death rate of only 7% for fBAV and for fBPV. Our prenatal technical results are comparable with the latest publication by Friedman et al. from Boston [12]—the procedures were technically successful in 101/123 (83%), with a higher technical success rate in the more recent procedures (94% vs. 73%). Our results seem to be much more favorable than the results published in the IFCIR (fetal survival 80% and technical success 81% [1]), and much better than a recently published national study (78.6% technical success and 32% procedure-related mortality) [13]. 

Major complications during fBAV occurred in cases with severe fibroelastosis of the left ventricle, in which thrombus formed in the ventricle following its puncture. Moreover, its function did not improve despite the successful opening of the aortic valve. As in those cases, pre–procedure echocardiography showed very low pressure in the left ventricle; according to recent publications those fetuses probably would no longer be scheduled for FCI [13]. 

We have shown that general anesthesia of the mother is not necessary to perform FCI procedures. As far as we know, we are the only center performing FCI in conscious maternal analgosedation, in contrast to protocols described by Tulzer et al. [8] where general anesthesia of the mother is a routine. 

In contrary to Schidlow et al. [14], who gave medications to the fetus through intramuscular or intracardiac routes, we used (in more than 98% of cases) the intraumbilical route, without any complications. Taking into account that access to the umbilical vein gives the possibility of both precise dosing of the medications and allows performing laboratory diagnostic tests, we assume that in experienced hands it could be the way of choice for fetal anesthesia for FCI. We think that intracardiac drug administration, which carries the risk of an additional damage of the ventricle wall, and consecutive pericardial bleeding should be reserved only for fetal resuscitation, when severe bradycardia prolongs the delivery time of the drugs from the umbilical vein to the heart. The intracardiac route was used by us only in fetuses who required immediate resuscitation due to bradycardia, which did not improve after fluid evacuation from the pericardium. 

According to our observations, blood must be evacuated from the pericardial cavity immediately to prevent fetal bradycardia and hemodynamic failure. If pericardial effusion persists, quick formation of the thrombus around the heart occurs, making its evacuation with the needle impossible. We observed that even very small amounts of blood (2–3 mL) in the pericardial cavity of a 20-week fetus significantly decreased chamber filling, causing cardiac insufficiency. Since we started to evacuate pericardial fluid immediately after needle withdrawal, instead of waiting for bradycardia and then giving adrenaline, the necessity of its administration became exceedingly rare, as the fetal heart rate normalized in most cases soon after fluid evacuation. It was not necessary to deliver any baby after the procedure due to hemopericardium or bradycardia, in contrast to what was described by Yoon et al. [15]. We also learned that it is very difficult to evacuate pericardial effusion by simply withdrawing the 18 G needle from the heart to the pericardial cavity after the FCI. It is mostly caused by an inappropriate (perpendicular) angle between the needle and the wall of the ventricle. Aspiration of the blood causes obstructing of the needle tip by the wall of the ventricle. With growing experience, we were always prepared with another needle (20–21 G) ready to use for decompressing the tamponade. This needle should be introduced into the pericardial cavity parallel to the ventricle wall immediately after diagnosis of pericardial bleeding to allow its sufficient emptying.

The use of a Y-connector while using a 17 G needle was a modification that proved very important because we realized that even excessive fetal bleeding flowing down on the surface of the needle might not have been easily noticed during the procedure.

Contrary to one of the most experienced centers [16], visualization of the fetal heart and heart puncture were performed by the same person (obstetrician). This is a typical way to perform most ultrasound-guided procedures in our center, and although it is sometimes very demanding, it enabled our obstetrician to gain precise control of the needle position during the entire procedure.

The anterior placenta was a factor limiting the area of the puncture site and the possibility of internal maneuvers with the needle to change fetal position. While inserting the needle through the placenta, one must be careful to avoid unintended damage of the umbilical insertion site and fetal vessels leading to it. Regarding the anterior placenta, needle movements should by extremely limited because of the risk of feto-maternal hemorrhage, bleeding from the placental surface, and abruption. 

Routine tocolysis was not necessary after FCI. Only in one case, in which fetal position was not optimal but urgent procedure was necessary, prolonged manipulation with the needle through the anterior placenta was performed in order to change the position of the fetus. The procedure was complicated with contractions and then placental abruption. 

In our material, in most cases FCI did not influence the time and mode of delivery. The median gestational age at delivery after fBAV, fBPV, and fBAS+ was 39 weeks. In cases of fetal atrioseptoplasty, the median gestational age at delivery was lower due to associated heart failure and polyhydramnios. In our hospital, neither previous prenatal cardiac intervention nor fetal cardiac defect were indications for cesarean section (CS), which was performed only due to obstetrical reasons. The rate of CS in our center was 40% of fBAV and 38% for all procedures, in contrast to 70% CS for all procedures and 67% CS for fBAV when the delivery was in another center.

## 5. Conclusions

We believe that FCI can be safely and effectively performed by an experienced team of obstetricians and cardiologists. In order to shorten the learning curve and minimize the risk of complications for the mother as well as for the fetus, any new team who wishes to start cardiac intervention should be first experienced in other fetal procedures and then should additionally undergo training in a cardiac fetal therapy center.

## 6. Limitations of the Study

FCI should not yet be considered as a standard treatment, as there are no current practice guidelines supporting its performance, but rather a very promising intervention offered to selected cases wishing to participate in research protocols, performed exclusively in centers of excellence in this field.

## Figures and Tables

**Table 1:** Technical aspects of all fetal cardiac procedures between June 2011 and April 2020.

Type of Interventional Procedure	Number of Fetuses/Number of Procedures	Median GA of the First Procedure	Success No./(%)	PE Drain No./(%)	PrR Deaths		PrNR Deaths	Brady No./%	Fetal Resuscitation Drugs No./%	Live Birth No/%	GA Delivery (Median)		Mode of Delivery CC/VD %	
					IUD	Postnatal	IUD				Our Center	Other Center	Our Center	Other Center
fBAV	88/94	25	84/89	59/63	6	1	3	23/24	15/16	77/90 †	39	38	25/37 40/60	10/5 67/33
fBPV	13/15	24	12/80	9/60	1	0	0	8/53	5/33	11/92 *	39	1 × 37 w. 1 × 33 w.	3/6 33/67	2/0 100/0
Atrial septoplasty (fBAS−)	5/5	24	4/80	4/80	1	0	0	3/60	2/40	4/80	1 × 29 w. 1 × 38 w.	1 × 29 w. 1 × 39 w.	0/2 0/100	1/0 100/0
Atrial stent (fBAS+)	14/14	27	12/86	8/57	2	0	0	3/21	1/7	12/86	37	33	4/7 36/64	1/0 100/0
Total	113/128				9	1	3			98/89	39	38	30/48 38/62	14/6 70/30
More than one procedure	14/29				1	0	0			12/92 *	39	34	4/8 33/67	1/0 100/0

Legend. fBAV: fetal balloon aortic valvuloplasty; fBPV: fetal balloon pulmonary valvuloplasty; GA: gestational age (weeks), IUD: intrauterine death; PrR: procedure-related; PrNR: procedure non-related; Brady: fetal bradycardia after the procedure; w.: weeks of delivery; CC: cesarean section; VD: vaginal delivery; * one pregnancy continues; † two pregnancies continue. Line “Total” is not the sum of previous lines, as there were 14 fetuses who had more than one procedure (8 fetuses had the same procedure twice, 5 fetuses had two different procedures, and 1 fetus had three different procedures).

## Supplementary Materials

The following are available online at https://www.mdpi.com/2077-0383/9/9/2888/s1, File S1: Technical aspects of all FCI, File S2: Technical aspects of fBAV, File S3: Atrial opening, File S4: fBPV technical aspects, Video S1: fBAV video, Video S2: Stent IAS video, Video S3: fBPV video.
