# Supplementary material for: Fetal Cardiac Interventions—Polish Experience from “Zero” to the Third World Largest Program
Source: J Clin Med. 2020 Sep 7;9(9):2888. doi: 10.3390/jcm9092888 (PMC7576494; doi:10.3390/jcm9092888)
Supplement: Supplementary file 1 [file jcm-09-02888-s001.zip › File S1 Technical aspects of all FCI.docx]

*Suppl. File 1 Technical aspects of all FCI*

*The protocol on preparing pregnant women for the fetal intervention:*

Before the procedure mothers were fasting, received intravenous hydration; an antibiotic was administered 30 minutes before the intervention (amoxicillin 1g with clavulanic acid 0,2g), as well as metoclopramide 10 mg iv and ranitidine 50 mg as a prevention of infection and aspiration of stomach contents in the mother.

*Equipment:*

All interventions (fBAV, fBPV, fBAS) were performed using 3 different ultrasound machines: Accuvix A30, probes: C2-61C, 2-6 MHZ; Samsung WS80A, probes CV1-8A, CA1-7A; 3 procedures were completed using an Epiq 7, probe C9-2. They were all recorded on DVDs or on external discs.

The team used 0,014" Abbott Hi-torque Whisper MS coronary guidewire and coronary balloons (Maverick or Sequent) which were introduced through the thinnest needle possible (usually 18G Cook trocar needle). In rare cases a 17G Argon Co-Axial needle had to be used. Other needles (20G and 21G) were ready for a pericardial puncture in case bleeding occurred.

FBS was performed with a 22 G needle.

Guidewires and catheters were washed with 0.9% NaCl solution before insertion into the needle for a better slip, we also added standard heparin (UFH) to this solution (at a dose of 1 UI per 1 ml of fluid).

*Medications:*

Before each procedure medications for fetal anesthesia and resuscitation: fentanyl, atracurium, atropine and adrenaline - were prepared in separate syringes.

Doses: Atracurium 0.2 mg/kg EFW, Fentanyl 20 mcg/kg EFW iv, adrenaline 10-30 mcg/kg EFW, atropine 20 mcg/kg EFW.

Volume expander, hydroxyethyl starch (HES/HAES), was also readily available in the operating theatre in case of excessive fetal bleeding. If blood components were necessary, they could be immediately delivered from the local hospital blood center.
