# Supplementary material for: Fetal Cardiac Interventions—Polish Experience from “Zero” to the Third World Largest Program
Source: J Clin Med. 2020 Sep 7;9(9):2888. doi: 10.3390/jcm9092888 (PMC7576494; doi:10.3390/jcm9092888)
Supplement: Supplementary file 1 [file jcm-09-02888-s001.zip › File S2 - BAV_technical aspects and complications.docx]

*Suppl. File 3 Technical aspects of fBAV*

*fBAV technique*

The obstetrician punctured the left ventricle through the apex, as close as possible to the ventricular septum to avoid damage of the mitral valve. The needle was placed just below the aortic valve. Guidewire was introduced through the stenotic valve by the invasive cardiologist. After fixating it in the aortic arch, the balloon was introduced. The size of the balloon was 1.1 to 1.2 of the valve diameter. The balloon was placed within the aortic valve and inflated three times. (Fig. 4) After that the balloon was pulled back through the needle, which was possible in 81 cases (86%).

*Technical results and complications of fBAV*

In one fetus in whom aortic valve atresia was documented after delivery neither guide wire, nor the balloon crossed the valve during two consecutive procedures. The diameter of the valve annulus was 3.5 mm, aortic valve leaflets were visible during fetal echo examination, left ventricle size was reasonably good, but color Doppler mimicked patency of the valve. It was the reason why we decided to repeat this procedure.

In 3 fetuses the first attempt was unsuccessful due to complications. In 2 severe fetal bradycardia occurred just after needle puncture, so the procedure was stopped before aortic valve could be crossed. Both fetuses recovered and next procedures were successfully carried on one and five weeks later respectively. In the third fetus the thrombus formed just after the LV puncture, it closed the needle which had to be exchanged and introduced again. After that a guidewire and a balloon were introduced, but severe thrombus persisted and additionally fetal bradycardia occurred, so dilatation of the valve was impossible. The fetus was then successfully resuscitated and the pregnant mother was discharged home according to the standards of care. At the monitoring visit one week later unexplained intrauterine fetal death was diagnosed. In 3 other fetuses the procedure of fBAV had to be repeated after 4-6 weeks due to restenosis of the aortic valve and it was successfully performed.

*Pericardial effusion and bradycardia* were the most common complications. Usually, the pericardial bleeding occurred after needle withdrawal from the heart and if not immediately successfully drained was then followed by bradycardia. In two cases pericardial effusion appeared just after the ventricular puncture causing severe fetal bradycardia. The needle had to be withdrawn to the pericardial cavity and the effusion was successfully drained. After this, the procedure was continued. In one case the procedure was postponed by 3 weeks due to thrombus in the left ventricular cavity. In the second fetus another needle was used to drain simultaneously fetal pericardium during the fBAV which was carried out effectively.

In 59 cases (63%) additional pericardial puncture was necessary to evacuate pericardial effusion. We always attempted to drain the blood with the 18G needle withdrawn from the left ventricle to the pericardial cavity, but in majority of cases it was not possible as the needle was perpendicular to the wall of the ventricle. An additional pericardial puncture with a 22G needle had to be performed parallel to the pericardial cavity, and only that enabled effective fluid evacuation. The amount of evacuated blood during one FCI ranged from 1 to 30 ml. In 13 fetuses bradycardia was treated with intracardiac adrenaline bolus. In one case adrenaline was given into the umbilical vein. In one case balloon rupture occurred in the left ventricular cavity, without visible complication to the fetus. Postnatally it was found that the aortic valve was arthretic.

*Thrombus formation* occurred in 6 cases, all of those fetuses had severe left ventricular fibroelastosis which proved to be difficult to perforate. Probably unintended detachment of the internal layer could cause the excessive activation of coagulation system. Since we started adding heparin into the solution in which guide-wires and catheters were flushed, manipulating within the needle was more smooth, however such procedure did not protect the intra cavity thrombus formation.

*Aortic insufficiency* after the procedure appeared in 52 fetuses: mild in 28 fetuses; in 3 of them it disappeared after 1 hour of observation; mild to moderate in 6 fetuses, moderate in 12 fetuses. Severe aortic insufficiency was created in 6 fetuses, their aortic valves were very dysplastic. In all of those fetuses the umbilical pulsatility index (PI) increased due to “the stealing effect”. One fetus with abnormal umbilical flow caused by severe aortic insufficiency died at 22 weeks, 3 days after the procedure. In this fetus left ventricle (LV) function was impaired and did not improve after the procedure. Opening the aortic valve caused a “functional aortic atresia” with severe “systolic” aortic valve insufficiency. In all other cases the umbilical artery PI normalized later in the pregnancy, together with decreasing of post-procedural aortic insufficiency

*Fetal deaths*

Procedure related death (PRD) occurred in 7 fetuses (7,4%) and the last one PRD was in 49^th^ procedure in 2015. Three of these fetuses had severe biventricular heart failure secondary to critical aortic stenosis, with left ventricular fibroelastosis and severe foramen ovale restriction. Right ventricle was compressed by enlarged left ventricle. In all of them immediately after left ventricular puncture thrombus appeared in the ventricular cavity, with severe bradycardia which did not respond to drug resuscitation. In the fourth fetus at 21 weeks fBAV caused functional aortic atresia, RV failure, placental insufficiency and finally fetal death. In the fifth fetus the procedure was complicated due to unfavorable fetal position. There were two attempts, the second was effective. After the procedure the fetus appeared to be in a good condition, except for low velocity flow in the umbilical and middle cerebral artery, which had already been observed before the operation. This fetus died during the first night after the procedure due to a thrombus formation in the pericardial cavity, so it is suspected that there was an additional issue with his condition. The sixth fetus died at the operating theater after seemingly uncomplicated and effective procedure – the reason was fetal bradycardia which did not respond to any treatment. In the seventh fetus fBAV was than carried out successfully but placental abruption was diagnosed 10 hours after the procedure. In this case emergency cesarean section was performed. The baby was born at 25 weeks of pregnancy and died shortly after delivery due to prematurity with and poor ventricular function.

Three other fetuses died in utero later in pregnancy, and it was not related to the procedure. Two mothers were heavy smokers and their fetuses died in 38 (6 weeks after fBAV) and 28 weeks of pregnancy. The third fetus died three weeks after the procedure due to unknown reason – this was a fetus with balanced translocation inherited from the mother.

Procedure-related deaths: 2/31 in the first group, 4/31 in the second, 0/32 in the third.

p for groups 1-2 = 0,67, p for groups 1-3 = 0,24, p for groups = 0,052 (borderline significant, yet has to be interpreted with caution, due to small numbers).
