# Supplementary material for: Fetal Cardiac Interventions—Polish Experience from “Zero” to the Third World Largest Program
Source: J Clin Med. 2020 Sep 7;9(9):2888. doi: 10.3390/jcm9092888 (PMC7576494; doi:10.3390/jcm9092888)
Supplement: Supplementary file 1 [file jcm-09-02888-s001.zip › File S3 - atrial opening.docx]

*Suppl File 4 – Atrial opening*

*Atrioseptoplasty and stent placement techniques*

The needle was introduced by the obstetrician through the fetal chest. She always punctured the anterior wall of the right atrium, then the interatrial septum and stopped in the left atrium. As in BAV she punctured the heart with the left hand, kept the transducer to monitor the procedure with the right hand. Then the cardiologist introduced the 0,014" guidewire which was stabilized in the pulmonary vein. The balloon catheter was introduced into the interatrial septum and pumped to its maximal diameter several times. In a case of stent placement instead of the balloon catheter, the coronary stent on the balloon was introduced through the needle. The stent was released when the septum was seen in the middle of it.

*Technical results and complications of fetal balloon atrioseptoplasty*

The first fetus died at night hours, after the standard monitoring was completed. The reason diagnosed post mortem was bleeding into the pericardial cavity. A very small hole created in the interatrial septum was found during autopsy. In the second fetus the interatrial septum was thick and despite several ballooning all created holes closed later during the pregnancy and the pulmonary vein flow did not change after this procedure. The third case was successful. It was a fetus with a floppy septum primum, so it could be effectively burst with the balloon. It was not possible to insert the stent due to the morphology of the interatrial septum. In the fourth case, the hole which was created during atrioseptoplasty closed spontaneously, so a stent was placed next day. It was a fetus after fBAV with severe fetal heart failure.

In one case we planned to insert the stent into the interatrial septum, but we changed the decision because visualization was very poor and safe insertion of the stent was technically impossible. We created a hole of about 3 mm diameter using a 17G needle. The pulmonary venous flow improved, blood stream was seen through the hole from the left to the right atrium. The baby was born at term and had a successful Blalock-Henlon operation.

*Technical results and complications of fetal* *stent placement in the interatrial septum*

There were two procedure related deaths. The first fetus died within 12 hours after fBAV performed the next day after stent placement in the interatrial septum. In the second case the position of one fetus unexpectedly slightly changed during the procedure as a result of maternal breathing movements. It caused minimal withdrawing of the needle so further correcting the position of the balloon and stent was impossible. The left atrium was very small and the stent damaged the wall of it, followed later by cardiac tamponade and fetal death within 24 hours. Five babies were born prematurely. One after fBAV, balloon atrioseptoplasty and stent placement; the reason was PPROM at 29 weeks (11 days after the last procedure) and the baby died few days after. The other patient delivered at 33 weeks in a regional hospital due to unknown reasons; without any consultation with the interventional team. Two cases of preterm births where due to preterm premature rupture of membranes in 30, 34 weeks respectively in patients with polyhydramnios. One preterm birth was in 36 weeks in a patient after excision of uterine septum one year before pregnancy.
