# Supplementary material for: Fetal Cardiac Interventions—Polish Experience from “Zero” to the Third World Largest Program
Source: J Clin Med. 2020 Sep 7;9(9):2888. doi: 10.3390/jcm9092888 (PMC7576494; doi:10.3390/jcm9092888)
Supplement: Supplementary file 1 [file jcm-09-02888-s001.zip › File S4 - fBPV_technical aspects and complications.docx]

*Suppl. File 6 - fBPV_technical aspects*

*fBPV technique*

The obstetrician punctured RVOT through the right ventricular anterior wall to place the needle just below the pulmonary valve. After that she pushed the needle across the pulmonary valve and removed the trocar. The cardiologist introduced a 0,014" Abbott Hi-torque Whisper MS guidewire with a balloon catheter. The balloon was 1.1 to 1.4 times larger than the pulmonary valve diameter. The balloon was inflated three times, after which it was pulled back into the needle whenever it was possible.

*fBPV compications*

One fetus died due to severe pericardial bleeding and after reviewing the recorded procedure it was suspected that the needle was inserted too high and probably damaged the pulmonary artery wall.

In another case the balloon ruptured when the pressure was about 4 atm and it was very difficult to remove it out of the fetal heart because it could not be introduced back into the needle. After removal of the needle and the damaged balloon the fetus started to bleed severely to the pericardium and required extensive resuscitation. We performed intensive fetal resuscitation with transfusion (to the umbilical vein and to the heart) of volume expanders, red blood cells, platelets, fresh frozen plasma and different medications. The fetus survived intact and the mother was discharged home safely after a few days following the procedure. The baby was born at term in a good condition.
